# Editorial: Challenges and Opportunities for Neuromuscular Disease Modelling Using Urine-derived Stem Cells

**DOI:** 10.3389/fphys.2022.848220

**Published:** 2022-03-07

**Authors:** Chaitra Sathyaprakash, Katsuhiko Kunitake, Yoshitsugu Aoki

**Affiliations:** Department of Molecular Therapy, National Institute of Neuroscience, National Center of Neurology and Psychiatry, Kodaira, Japan

**Keywords:** urine-derived stem cells, human disease models, skeletal myotubes, muscle, transdifferentiation

Restoration of dystrophin function in muscle via exon-skipping antisense oligonucleotides (ASO) has been long heralded as a promising therapeutic strategy to treat Duchenne muscular dystrophy (DMD). The existence of over 1,800 *DMD* mutations in the form of deletions and duplications has made this disease very challenging to generate models and perform screening studies to identify the most efficient exon-skipping therapies for clinical trials. However, increasing discoveries of several other cellular disease mechanisms associated with DMD have increased the scope for targeted treatment and the need for patient-specific DMD myogenic models to screen. Recent studies in *Frontiers of Physiology* identify several secondary cellular phenotypes in murine models of muscular dystrophy (Dong et al.; Valentine et al.), which may serve as essential targets in combinatorial therapies in DMD patients. While these mouse models are applicable as surrogates for human diseases, human-specific pre-clinical studies are crucial to examine the effects on individual patients.

In recent years multiple studies have suggested urine-derived stem cell (USC) cultures as a solution to overcome the challenge of obtaining somatic cells from human patients. A screening study using RNA sequencing and protein profiling via immunofluorescence and immunoblotting demonstrated that USCs readily express 571 genes that have been implicated in 16 neuromuscular diseases (NMD) (Falzarano et al., 2021). To confirm whether USCs model relevant cell lineages, changes in gene expression was examined in USCs and MyoD-induced myogenic cells from 3 non-disease individuals. Differential expression analysis of the mRNAs expressed in these cells, following bulk RNA transcriptomics, showed that 154 genes associated with NMDs were enriched in the myogenic cells compared to USCs. Conversely, 127 genes were enriched in USCs compared with the myogenic cells. Furthermore, 165 genes associated with NMDs were not expressed in the myogenic cells. A number of genes not expressed in USCs were switched on followed MyoD-induction, though their expression at the protein level was not tested. These included *DES, SGCD, SGCA, DTNA, MYL1* and *STAC3* (Figure 1). Similarly, a number of genes not usually expressed in the myogenic cells were downregulated following MyoD-induction from USCs, including *COL6A*. Significantly, the *DMD* gene was not expressed in either USCs or the induced myogenic cells, despite the authors previously demonstrating dystrophin protein's presence in similar cultures (Falzarano et al., 2016). Therefore, further optimisation for the time of analysis after this conversion is desired prior to establishing USC-myotubes as a model for NMDs. Alternative protocols using retroviral transduction of lineage determining transcription factor, MyoD1 combined with a histone methyltransferase inhibitor, 3- deazaneplanocin A hydrochloride (DZNep) in primary USC cultures also generates pure myotube cultures, expressing myogenin, followed by increasing levels of myosin heavy chain-2 and dystrophin (Takizawa et al., 2019). Furthermore, using this model Takizawa et al. (2020) demonstrated highly successful exon 23-skipping in myotubes derived from DMD patient USCs using ASO transfection, showing restored levels of dystrophin mRNA and protein. Overall, using the method of DMD patient- derived USC-myotube direct differentiation largely increases the efficiency of obtaining human cellular models of any DMD mutation and provides a robust screening platform for exon-skipping therapies, which are likely to benefit pre-clinical trials for DMD, and indeed any neuromuscular or neurodegenerative diseases.

**Figure 1:**
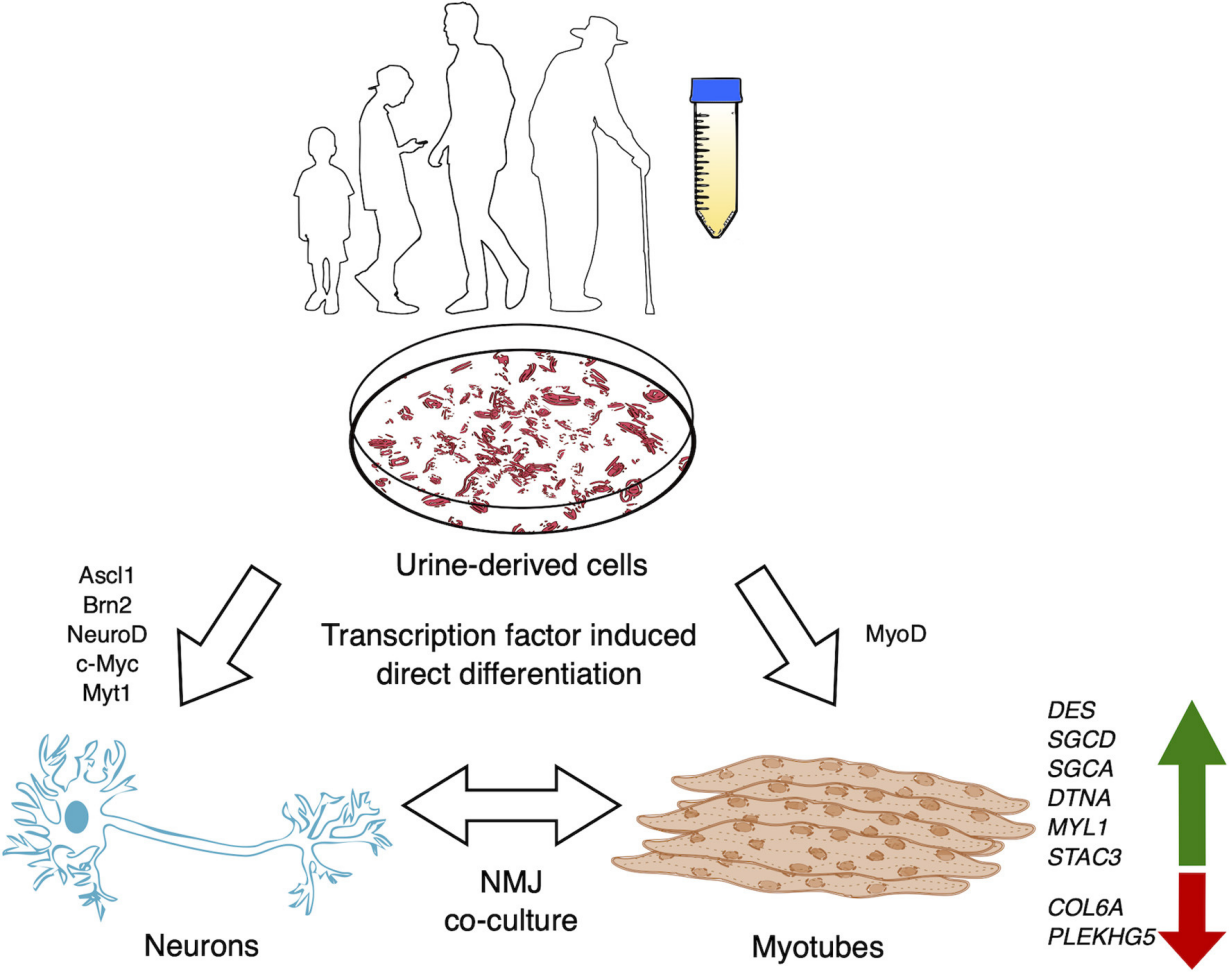
USCs may be isolated from the voided urine of patients with neurodegenerative or neuromuscular disease and non-disease individuals of any age or debilitating condition. USCs may be utilized in a myriad of ways, including the potential to derive directly induced cellular models (myotubes, motor neurons or co-cultures). Zhang et al. (2016) performed direct conversion of USCs into neuronal cells via exogenous expression of Ascl1, Brn2, NeuroD, c-Myc, and Myt1l, while myotubes may be *myodifferentiated* via MyoD expression. Genes implicated in NMDs are upregulated in USC-myotubes, making them a promising model for human neuromuscular diseases.

Increasing research establishing USCs as a faster, cheaper, rapidly proliferating, and contaminant-free source of cells has opened the doors for the opportunity to generate human cell models from the voided urine of the most vulnerable individuals, including DMD pediatric cases or late-stage amyotrophic lateral sclerosis patients. Protocols to transdifferentiate USCs into neuronal cells has been demonstrated, though high yield and expression of specific markers for neuronal subtypes has yet to be achieved (Zhang et al., 2016; Xu et al., 2019; Liu et al., 2020) (Figure 1). Meanwhile, fibroblasts can be directly converted to motor neurons with a yield of 66.4% ISL1^+^/Hoechst^+^ and >90% ISL1^+^/TUJ1^+^ (Tang et al., 2017). Regulating epigenetic states of USCs via additional compounds, like DZNep promotion of USC-*myodifferentiation*, to modulate *neurodifferentiation* for direct reprogramming should significantly benefit the study of adult-onset diseases in addition to DMD.

DMD patients will likely require the therapeutic intervention of secondary pathways altered in disease to restore full muscle integrity and function. For example, prior analysis of post-mortem human samples from DMD patients exhibited altered levels of specific fatty acids and phosphatidylcholine (PC) in the muscle cell membrane. Recent lipidomics data from dystrophic *mdx* mice revealed similarly altered ratios of PC 34:1 and 34:2, including some differences absent in humans (Valentine et al.). Due to such differences between the species, patient-derived USC-myotubes cultures revealing the cellular mechanisms that lead to alterations in human tissue may be crucial. This includes patient- specific differences in onset, progression and severity in lipid membrane composition and screening for therapies to restore non-disease lipid levels. Similarly, USC-myotubes may be used to examine the efficacy of metformin treatment on muscle function by examining sarcolemma integrity across large cohorts of patient-derived USC-myotube cultures, which was shown to improve muscle strength in mice (Dong et al.).

USCs may be robustly harvested from individuals of any age or debilitating condition, significantly broadening the cohort of patients accessible to study. In the case of neuromuscular diseases like DMD, the potential for these cells to be used for pre-clinical therapeutic screening, modeling understudied cognitive disease mechanisms and regenerative therapies, is highly optimistic. However, another significant challenge in the field lies in the efficiency of exon-skipping ASOs to be targeted to muscle tissue *in vivo*. At present, this remains outside the scope that USC-derived cultures may provide. Nevertheless, the authors feel that USCs are likely to become increasingly used as a model to study neuromuscular and neurodegenerative diseases, with several promising uses.

## Author Contributions

CS: writing—original draft. KK and YA: writing—review and editing. All authors contributed to the article and approved the submitted version.

## Conflict of Interest

The authors declare that the research was conducted in the absence of any commercial or financial relationships that could be construed as a potential conflict of interest.

## Publisher's Note

All claims expressed in this article are solely those of the authors and do not necessarily represent those of their affiliated organizations, or those of the publisher, the editors and the reviewers. Any product that may be evaluated in this article, or claim that may be made by its manufacturer, is not guaranteed or endorsed by the publisher.
